# Supplementary material for: Systems immunology integrates the complex endotypes of recessive dystrophic epidermolysis bullosa
Source: Nat Commun. 2025 Jan 14;16:664. doi: 10.1038/s41467-025-55934-7 (PMC11733305; doi:10.1038/s41467-025-55934-7)
Supplement: Supplementary file 1 — Supplementary Information [file 41467_2025_55934_MOESM1_ESM.pdf]

Systems immunology integrates the complex endotypes of recessive dystrophic epidermolysis bullosa

Nell Hirt[1†], Enzo Manchon[1†], Qian Chen[2], Clara Delaroque[3], Aurelien Corneau[4], Patrice Hemon[5], Safaa Saker-Delye[6], Pauline Bataille[7], Jean-David Bouaziz[1,7], Emmanuelle Bourrat[7], Alain Hovnanian[8], Helene Le Buanec[1], Fawzi Aoudjit[9], Hicham El Costa[10], Nabila Jabrane-Ferrat[10#], Reem Al-Daccak[1#*]

†These authors contributed equally.

# These authors equally contributed and jointly supervised this work.

Correspondence and requests for materials should be addressed to:

*Reem Al-Daccak, PhD

Hôpital Saint-Louis, Inserm U976

1, Avenue Claude Vellefaux, 75010, Paris - France

email: reem.al-daccak@inserm.fr

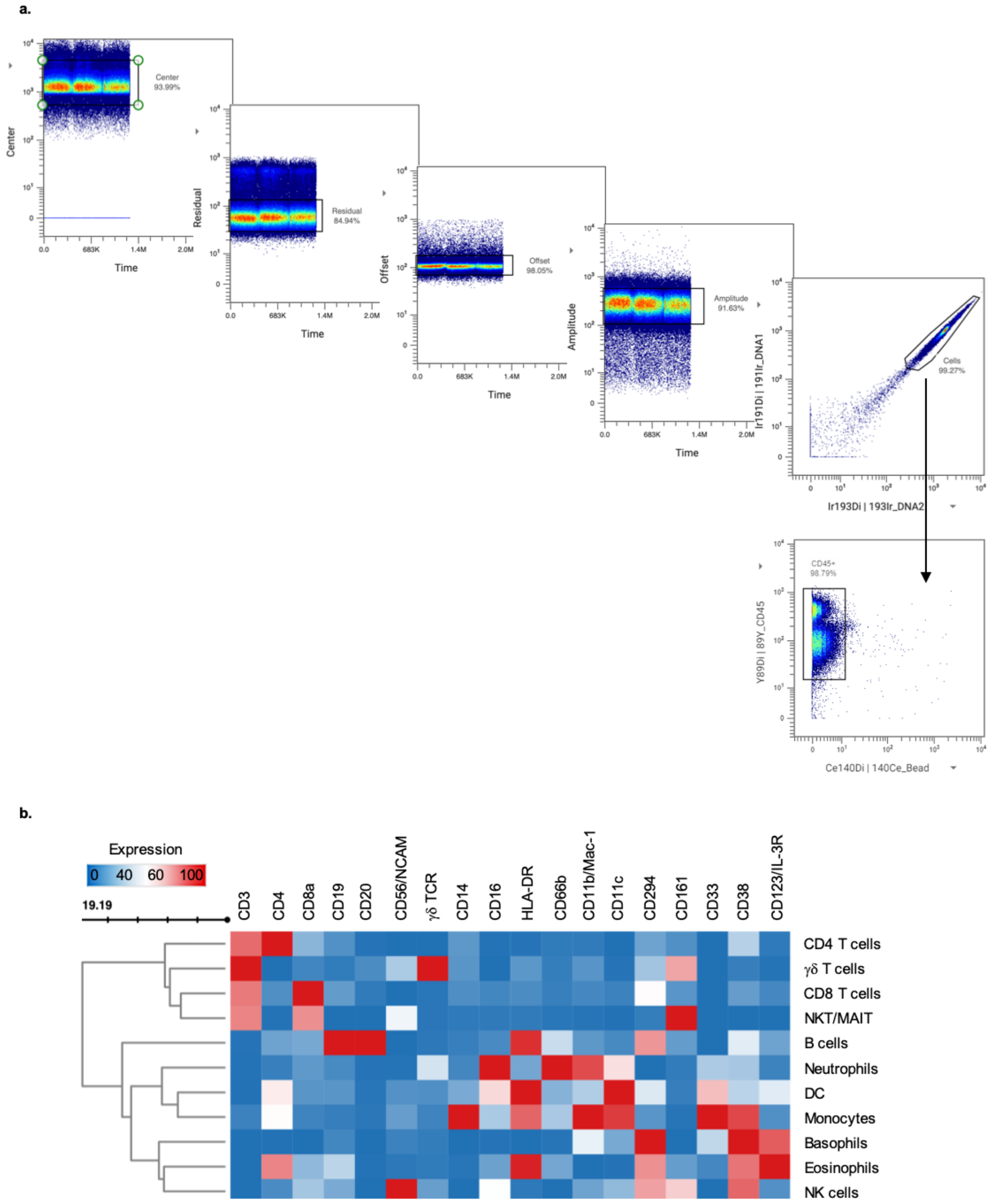

Supplementary Figure 1: Clustering of whole blood leukocytes. a. CYTOF gating strategy of whole blood cells showing the initial population of circulating white blood cells. A fixed number of 500,000 CD45-positive circulating immune cells were acquired for each patient and control to avoid any eventual bias in the analyses. **b.** Heatmap of 18 normalized marker expression of 105.10^3 cells/sample. PhenoGraph clustering of a total of 21 whole blood samples, 9 healthy controls and 12 RDEB adults.

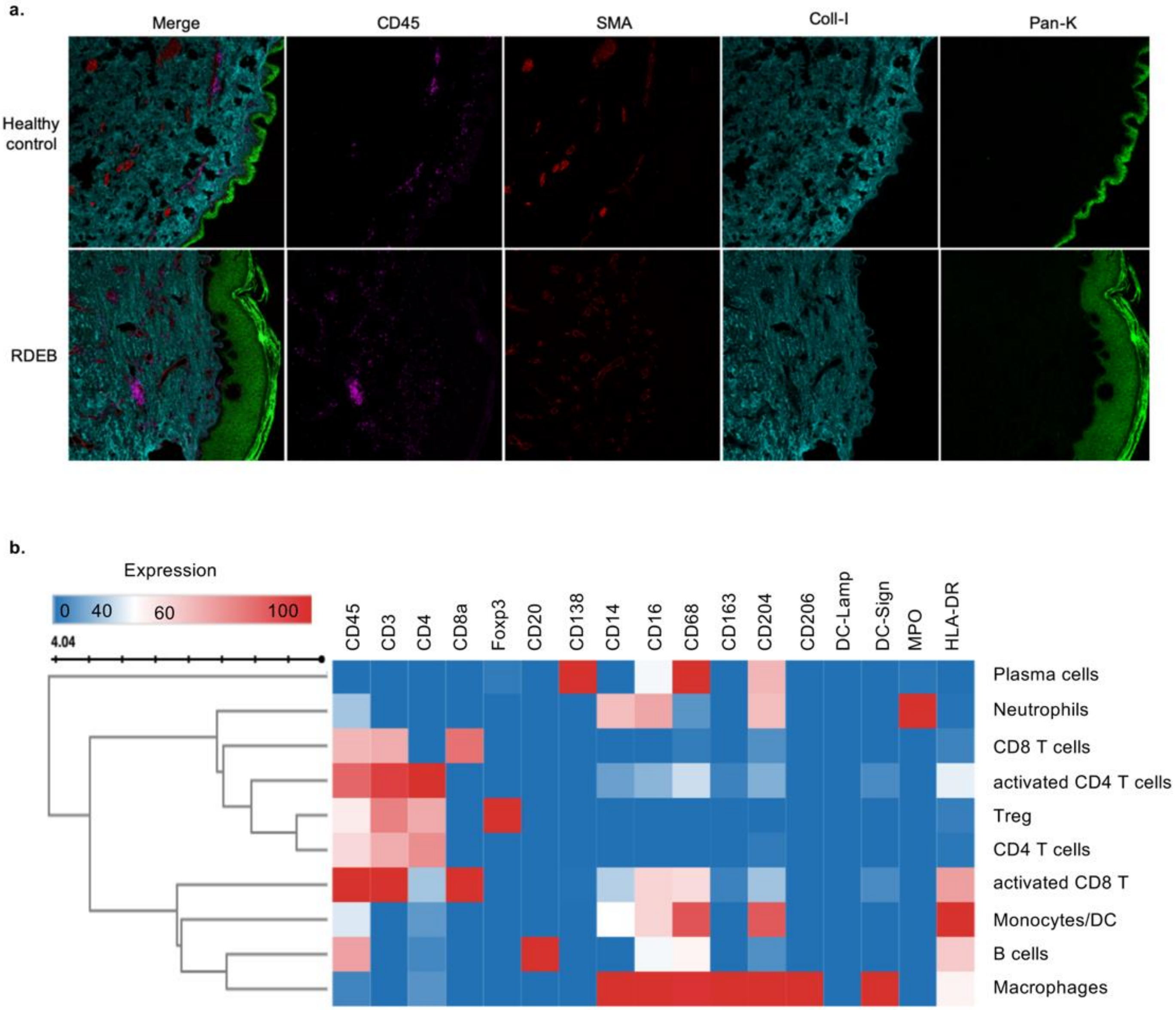

Supplementary Figure 2: a. Representative visualization (of three independent experiments) of structural, stromal, immune infiltrates and vascular components in skin biopsies by spatial mass cytometry-based single-cell imaging. Merge: panKeratin (green)/SMA (α-SMA) (red)/immune cell infiltrate ($CD45^+$, magenta)/Collagen type I (Coll-I, cyan). Enlarged images depicting the above. Skin biopsies from two healthy donors and skin biopsies form two RDEB patients showed similar results. **b.** Heatmap of 17 normalized marker expression. PhenoGraph clustering of 4 samples, 2 healthy controls and 2 RDEB adults.

a.

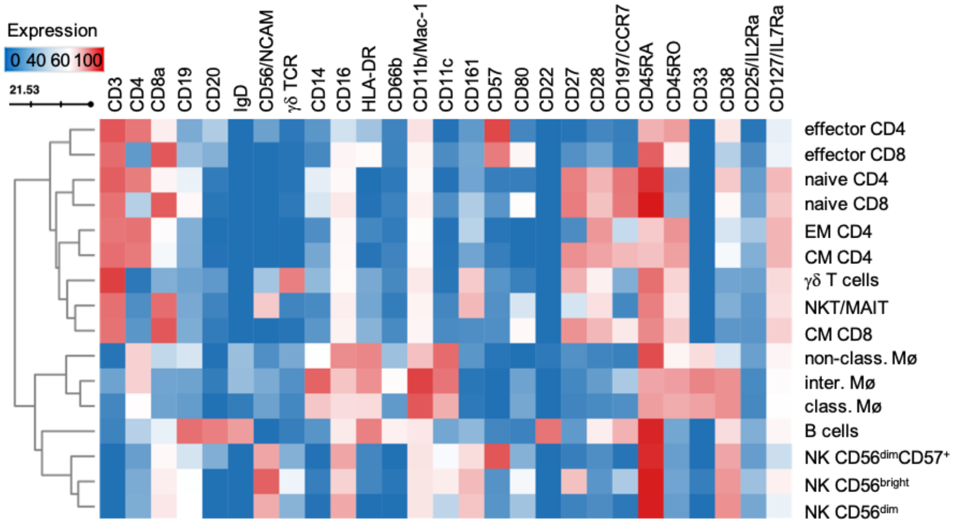

b.

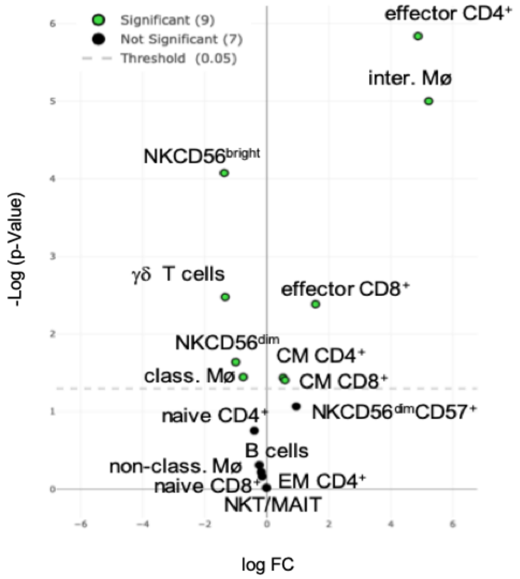

Supplementary Figure 3: Identification of adaptive and innate immune cell populations in RDEB and healthy control. a. Heatmap of 27 normalized marker expression of 25.10^3 PBMC/sample. PhenoGraph clustering of a total of 21 PBMC samples (9 healthy controls and 12 RDEB adults). **b.** EdgeR-based Volcano plot presenting differentially increased (right) and decreased (left) adaptive and innate immune cell populations in RDEB adults compared to healthy controls. The horizontal dashed line indicates significant threshold at p-value = 0.05.

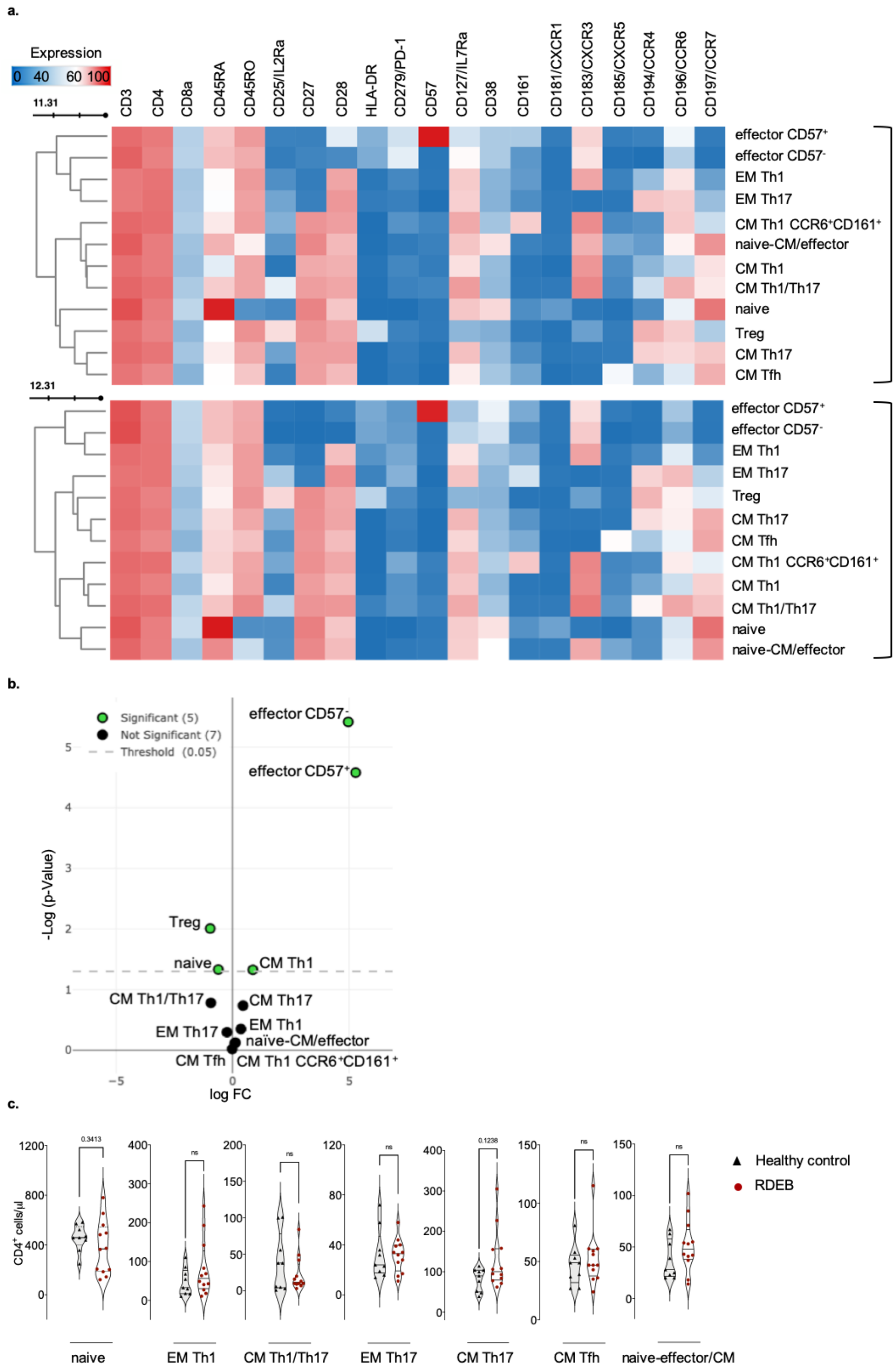

Supplementary Figure 4: Identification of CD4$^+$ T cell subsets in RDEB and healthy controls. a. Heatmap of 20 normalized marker expression of 8.10^3 CD4$^+$ cells/sample. PhenoGraph clustering of 9 healthy controls and 12 RDEB adults. **b**. EdgeR-based Volcano plot presenting differentially increased (right) and decreased (left) CD4$^+$ T cell subsets in RDEB adults compared to healthy controls. The horizontal dashed line indicates significant threshold at a p-value = 0.05. **c**. Violin plots comparing the absolute counts (cells/µl) of CD4$^+$ T cell subsets from 9 healthy controls (black triangles) and 12 RDEB adults (red circles) with median values presented by solid dark line. Statistical analysis is performed with two-sided unpaired *t*-test. Source data are provided as a Source Data file.

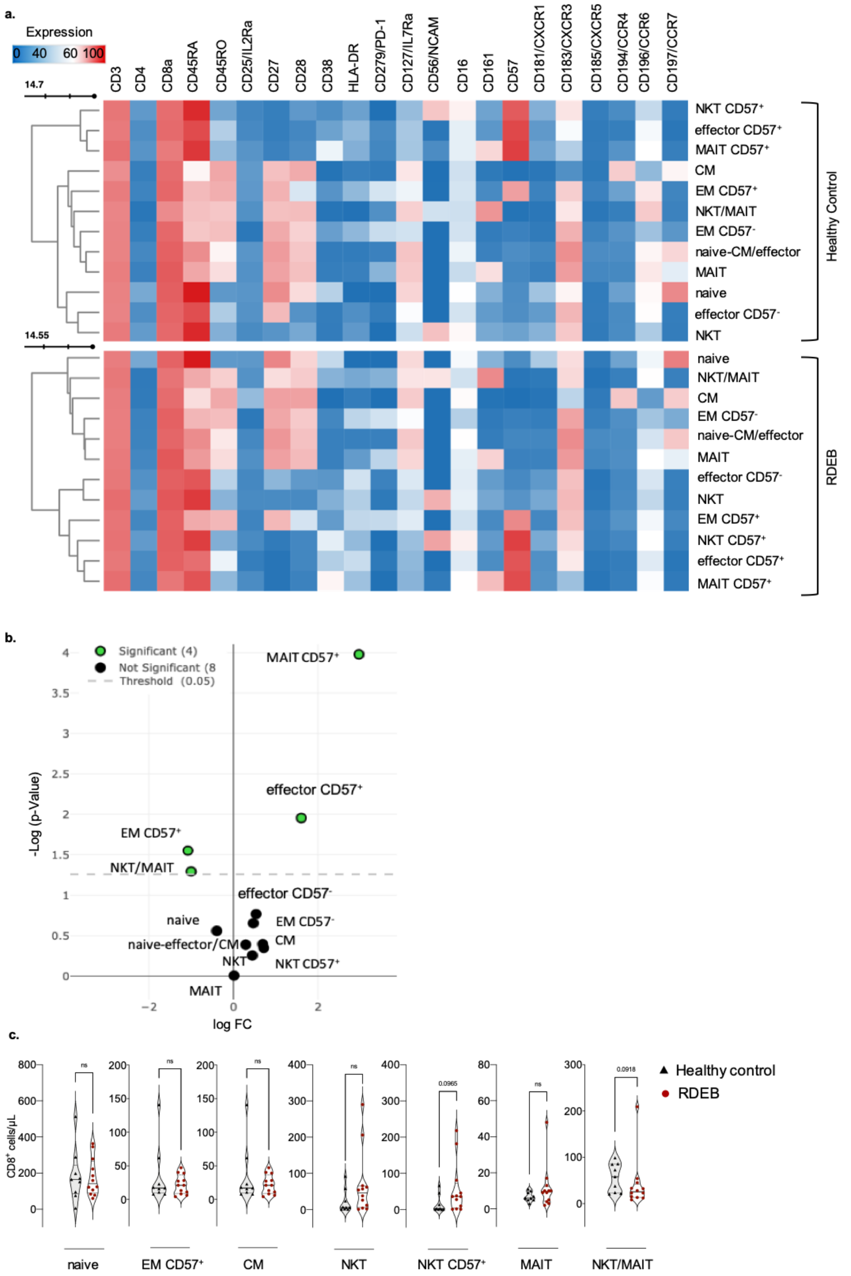

Supplementary Figure 5: Identification of CD8+ T cell subsets in RDEB and healthy controls. a. Heatmap of 22 normalized marker expression of 4.10^3 CD8$^+$ cells/sample. PhenoGraph clustering of 9 healthy controls and 12 RDEB adults. **b.** EdgeR-based Volcano plot presenting differentially increased (right) and decreased (left) of CD8$^+$ T cell subsets in RDEB adults compared to healthy controls. The horizontal dashed line indicates significant threshold at a p-value = 0.05. **c**. Violin plots comparing the absolute counts (cells/μl) of CD8$^+$ T cell subsets from 9 healthy controls (black triangles) and 12 RDEB adults (red circles) with median values presented by solid dark line. Statistical analysis is performed with two-sided unpaired *t*-test. Source data are provided as a Source Data file.

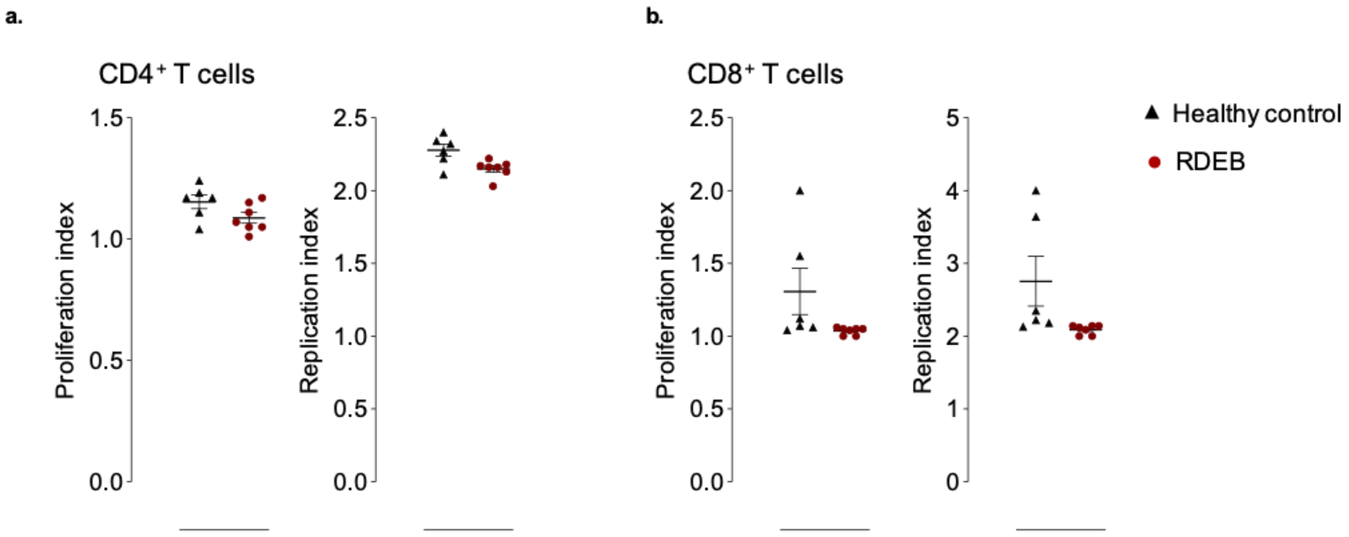

Supplementary Figure 6: Healthy controls and RDEB adults T cells proliferation at steady state. PBMC proliferation gated on **a.** $CD4^+$ and **b**. $CD8^+$ cells. Replication and Proliferation index (RI, PI respectively) are presented as scatter plots. Data are presented as mean values ± SEM from 6 healthy controls and 7 RDEB adults. Healthy controls (black triangles) and RDEB adults (red circles). Statistical analysis is performed with two-sided unpaired *t*-test.

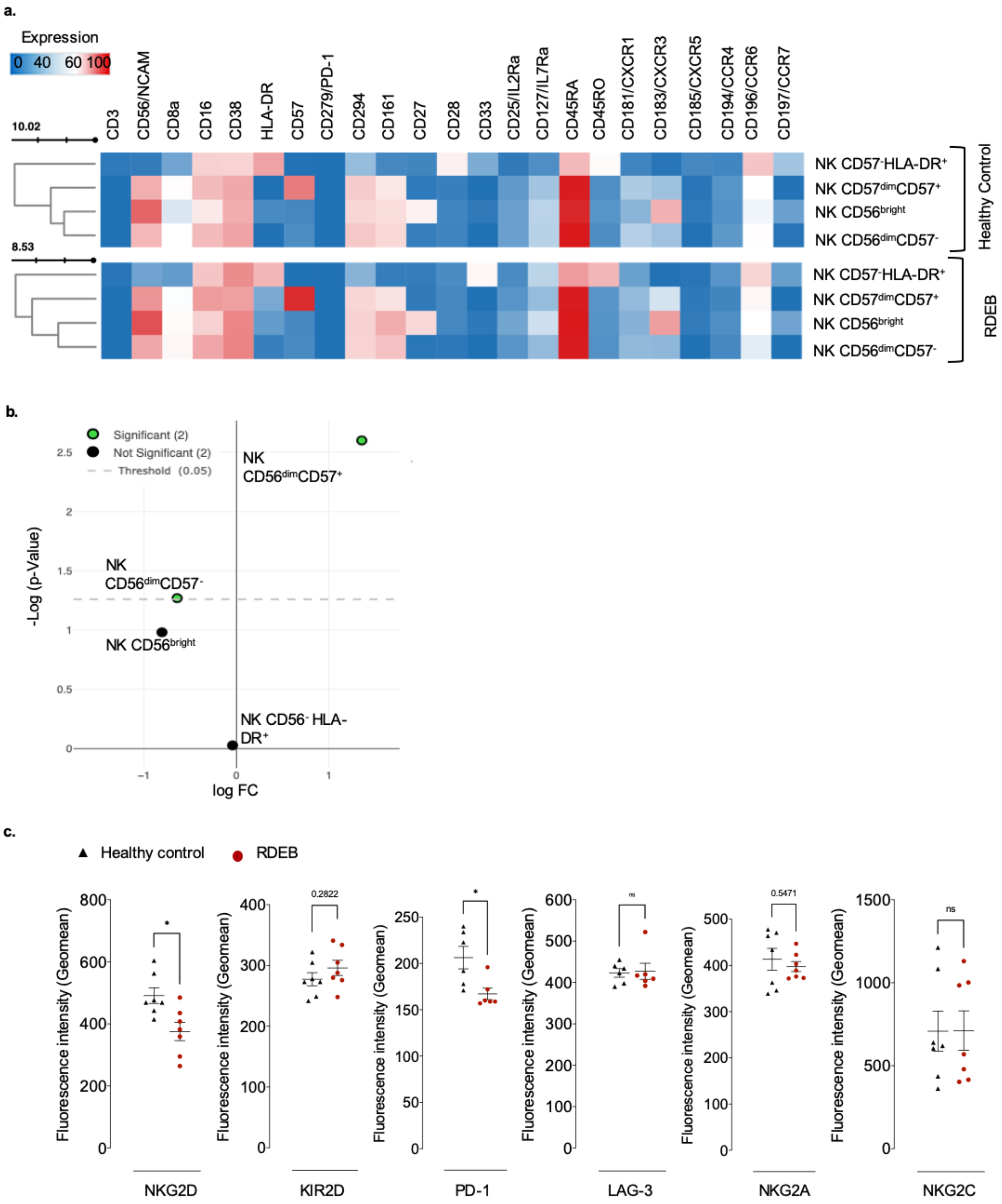

Supplementary Figure 7: Identification of NK cell subsets in RDEB and healthy controls. a. Heatmap of 23 normalized marker expression of 10^3 NK cells/sample. FlowSOM-based clustering of 9 healthy controls and 12 RDEB adults. **b**. EdgeR-based Volcano plot representing differentially increased (right) and decreased (left) of NK cell subsets in RDEB adults compared to healthy controls. The horizontal dashed line indicates significant threshold at a p-value= 0.05. **c**. Expression level (geometric mean fluorescence intensity) of activating and inhibitory NK cell receptors, and exhaustion- and senescence-related markers in RDEB adults (red circles) compared to healthy controls (black triangles). Data are represented as mean values ± SEM from 7 healthy controls and 7 RDEB adults (NKG2D, KIR2D, NKG2C, NKG2A), from 6 healthy controls and 6 RDEB adults (PD-L1 and LAG-3). Statistical analysis is performed with two-sided unpaired *t*-test. Asterisks represent the significant differences between RDEB adults and healthy controls (*p<0.05).

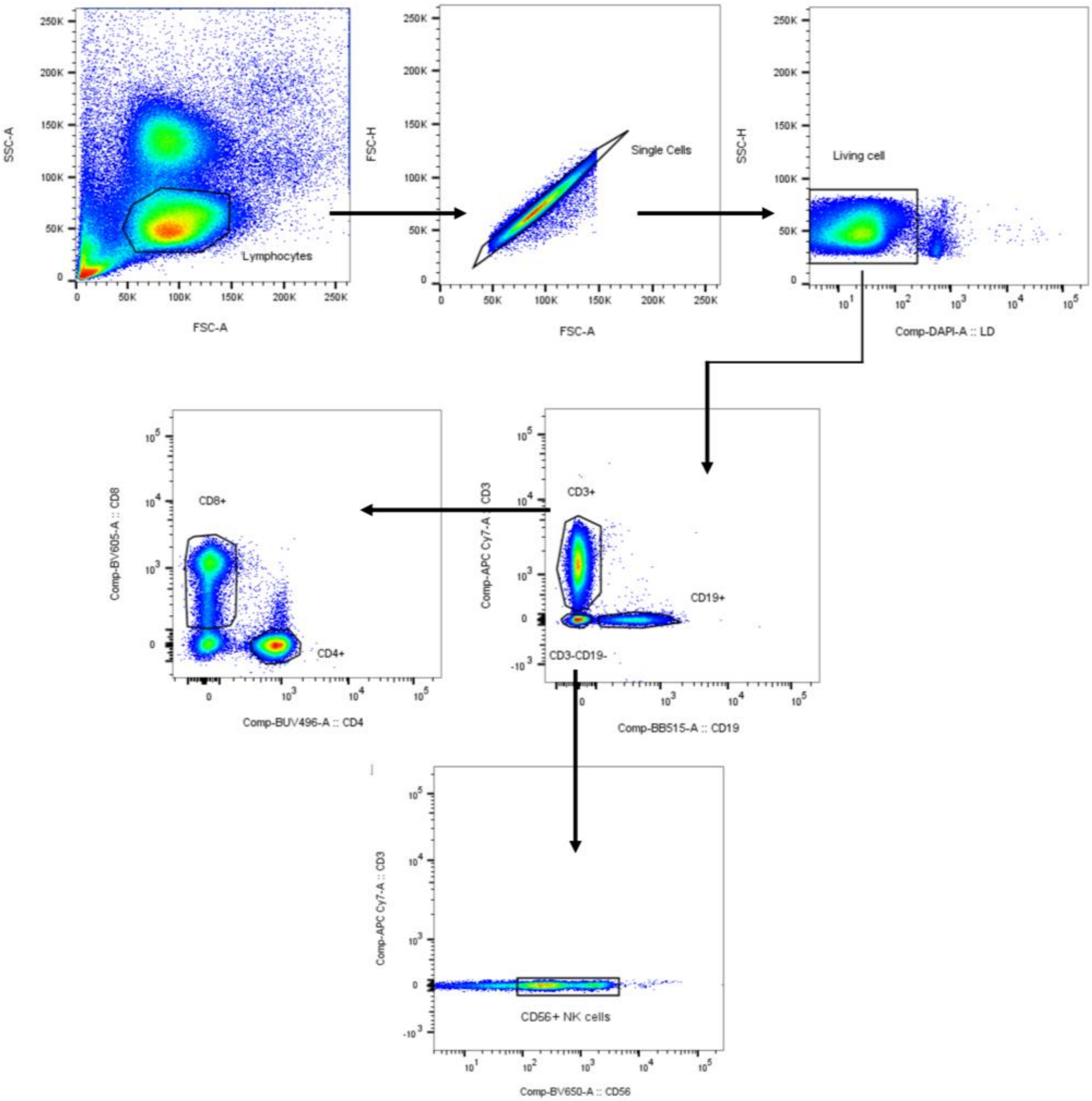

Supplementary Figure 8: SCENITH Gating strategy: SCENITH gating scheme to interrogate T and NK for surface markers and metabolism. Single, live lymphocytes in total PBMC were gated sequentially before division into $CD19^+$ B cells, $CD3^+CD4^+$ and $CD3^+CD8^+$ T cells, and $CD19^-CD3^-CD56^+$ NK cells.

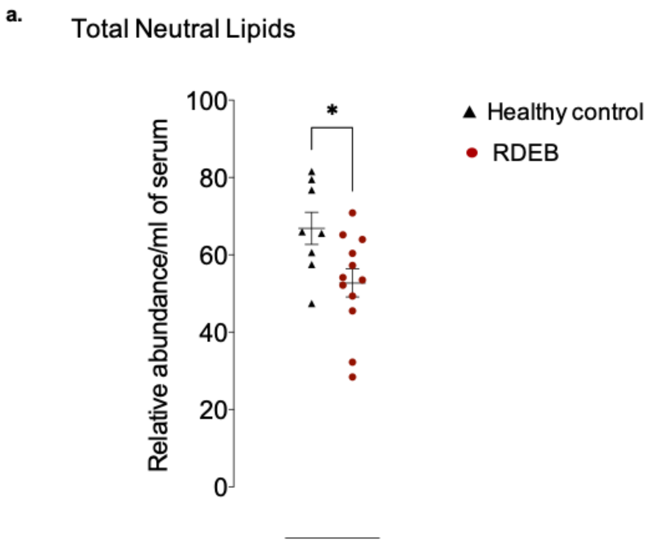

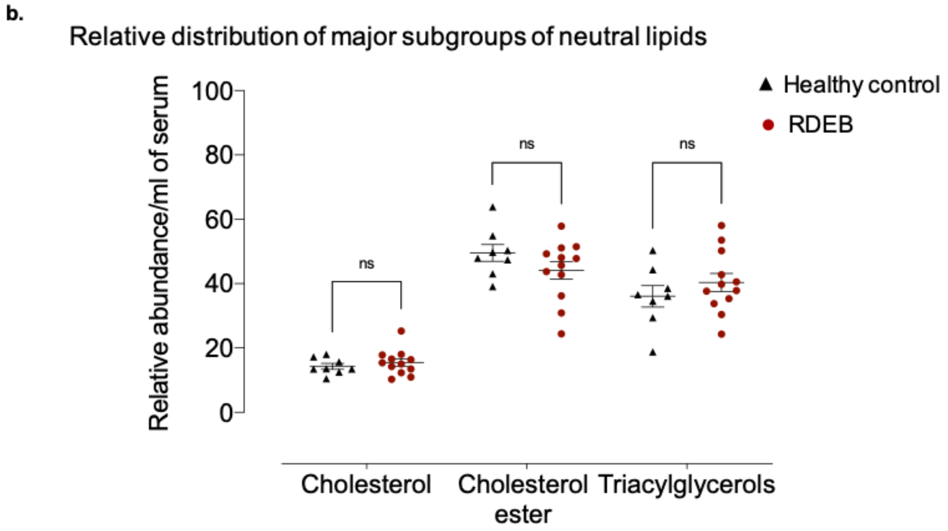

Supplementary Figure 9: Neutral lipids profile in RDEB adults. Relative abundance of total neutral lipids (**a**) and relative distribution of major subgroups of neutral lipids (**b**) in sera from 8 healthy controls and 12 RDEB adults. All results are presented as mean values ± SEM in scatter plots. Healthy controls (n=9) (black triangles) and RDEB adults (n=12) (red circles). Statistical analysis is performed with two-sided unpaired *t*-test. Asterisks represent the significant differences between healthy controls and RDEB adults (*p<0.05). Source data are provided as a Source Data file.

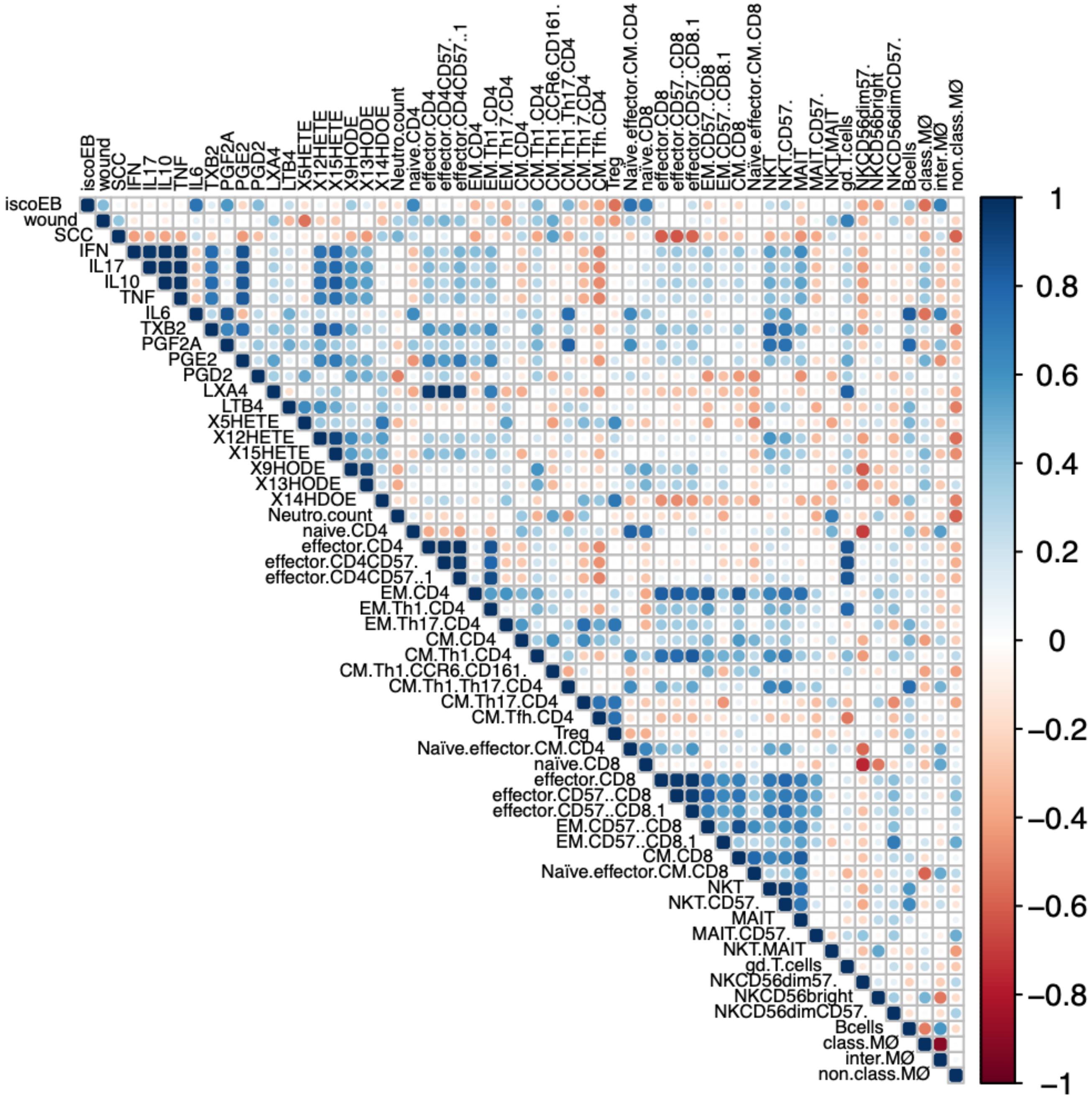

Supplementary Figure 10: RDEB correlation matrix. Correlation matrix based on clinical IscorEB, % wound area, SCC history, abundance level of cytokines, lipids, and the absolute counts of various immune cell subsets in RDEB adults. Matrix is colored according to correlation coefficient value, blue presents positive correlation, red presents negative correlation. Correlation matrix was computed using R (R version 4.1.2, package corrplot 0.92) and significance (p-value) is presented by the size of the circles where larger circles present higher significant correlation.

Supplementary Table 1: *COL7A1* pathologic variants of the 12 enrolled RDEB adults

Patient	*COL7A1* mutation	mutations (c.) (NM_000094.4)	mutations (p.)	consequences	Location exon/intron	rs	Domain	Ref PMDI
BM443	Homozygote	5499C>T	Gly1833Gly	Splicing	Exon 65	rs758886532	Triple Helices	19681861
YZ319	Homozygote	6187C>T	Arg2063Trp	Missense	Exon 74	rs121912849	Collagenous	9326325
CB523	Heterozygote	4027C>T	Arg1343*	Non-Sens	Exon 35	rs761234904	Triple Helices	8037202
		6146G>A	Gly2049Glu	Faux Sens	Exon 74	rs1410793870	Triple Helices	9326325
MB070	Homozygote	189del	Leu64Trpfs*40	Frame Shift + PTC	Exon 3	rs1381975764	von Willebrand factor, type A	23769655
GM990	Homozygote	2005C>T	Arg669*	Non-Sens	Exon 16	rs780261665	Fibronectin, type III	9881948
IHB706	-	-	-	-	-	-	-	-
KN769	Homozygote	267-3C>G	ND	Splicing	Intron 3	rs1559441291	ND	9326325
BA525	Heterozygote	4018C>T	Arg1340*	Non-Sens	Exon 35	rs761927109	Triple Helices	16189623
		7723G>A	Gly2575Arg	Faux Sens	Exon 104	rs760891216	Triple Helices	9326325
CT699	Heterozygote	431_434dup	Ile145Metfs*36	Frame Shift + PTC	Exon 5	No external annotation	von Willebrand factor, type A	12735646
		4899+1G>A	ND	Splicing	Intron 52	rs766168993	ND	15807692
DC622	Heterozygote	1067del	Tyr356Serfs*3	Frame Shift + PTC	Exon 9	No external annotation	Fibronectin, type III	ND
		2561dup	Pro855Thrfs*11	Frame Shift + PTC	Exon 20	No external annotation	Fibronectin, type III	ND
TC062	Homozygote	7012C>T	Arg2338*	Non-Sens	Exon 91	rs745874032	Triple Helices	10367729
BS190	Homozygote	6508C>T	Gln2170*	Non-Sens	Exon 81	rs1461012195	Triple Helices	16484981

Supplementary Table 2: Clinicopathologic characteristics of the 12 enrolled RDEB adults. IscorEB: Instrument for Scoring Clinical Outcome of Research for Epidermolysis Bullosa; BMI: Body Mass Index; SCC: Squamous cell carcinoma.

Patient	Sex	Age	RDEB subtypes	% Wound Area	IscorEB	BMI (kg/m^2)	Fibrotic Damage	Carcinoma History
BM443	Male	52	Localized	20	33	18.6	Esophageal stenosis (dilatation)	Moderately infiltrative & micro-invasive SCC
YZ319	Female	44	Intermediate generalized	30	60	16.4	Esophageal stenosis Synechiae hand	None
CB523	Male	49	Severe generalized	80	72	12.6	Esophageal stenosis Synechiae hand and foot	Invasive SCC
MB070	Female	33	Severe generalized	50	87	17	Esophageal stenosis (gastrostomy, dilatation) Synechiae hand and foot	Invasive SCC
GM990	Female	32	Severe generalized	80	77	19.6	Synechiae hand and foot Esophageal stenosis	Multiple SCC including invasive
IHB706	Male	35	Intermediate generalized	20	62	12.2	Esophageal stenosis (gastrostomy) Synechiae hand and foot	None
KN769	Female	32	Severe generalized	80	72	22.5	Esophageal stenosis (gastrostomy, dilatation) Synechiae hand and foot	Invasive SCC
BA525	Male	37	Severe generalized	30	70	14.3	Superior Esophageal stenosis (dilatation) Synechiae hand and foot	Invasive SCC
CT699	Female	26	Severe generalized	50	70	19.2	Esophageal stenosis Synechiae hand and foot	Invasive SCC
DC622	Female	36	Severe generalized	60	93	16.9	Esophageal stenosis (dilatation) Synechiae hand and foot	SCC
TC062	Female	31	Severe generalized	60	106	15.6	Esophageal stenosis Synechiae hand and foot	None
BS190	Female	25	Severe generalized	55	133	15	Synechiae hand and foot Esophageal stenosis (gastrostomy) Anal stenosis	Invasive SCC

Table 3: Mass cytometry (CYTOF) antibody information. A list of Standard Bio Tool Maxpar® Direct™ Immune Profiling Assay™ and the Maxpar® Direct™ Myeloid and B cell Expansion Panel 1 antibodies used in the study.

Supplementary Table 3: Lineage-specific metal-tagged antibody panel for single-cell mass cytometry (CyTOF)

Metal_Target (Clone)	Whole blood UMAP - PhenoGraph	PBMC UMAP - PhenoGraph	CD4 T cell opt-SNE - FlowSOM	CD8 T cell opt-SNE - FlowSOM	NK cell opt-SNE - FlowSOM
89Y_CD45 (HI30)	89Y_CD45 (HI30)	89Y_CD45 (HI30)	89Y_CD45 (HI30)	89Y_CD45 (HI30)	89Y_CD45 (HI30)
141Pr_CD196/CCR6 (G034E3)	170Er_CD3 (UCHT1)	170Er_CD3 (UCHT1)	170Er_CD3 (UCHT1)	170Er_CD3 (UCHT1)	170Er_CD3 (UCHT1)
142Nd_CD181/CXCR1 (8F1/CXCR1)	145Nd_CD4 (RPA-T4)	145Nd_CD4 (RPA-T4)	145Nd_CD4 (RPA-T4)	145Nd_CD4 (RPA-T4)	163Dy_CD56/NCAM (NCAM16.2)
143Nd_CD123 (6H6)	146Nd_CD8a (RPA-T8)	146Nd_CD8a (RPA-T8)	146Nd_CD8a (RPA-T8)	146Nd_CD8a (RPA-T8)	146Nd_CD8a (RPA-T8)
144Nd_CD19 (HIB19)	163Dy_CD56/NCAM (NCAM16.2)	171Er_CD20 (2H7)	149Sm_CD45RO (UCHL1)	149Sm_CD45RO (UCHL1)	148Nd_CD16 (3G8)
145Nd_CD4 (RPA-T4)	151Eu_CD161 (HP-3G10)	144Nd_CD19 (HIB19)	150Nd_CD45RA (HI100)	150Nd_CD45RA (HI100)	161Dy_CD38 (HB-7)
146Nd_CD8a (RPA-T8)	164Dy_TCRgd (B1)	174Yb_IgD (IA6-2)	153Eu_CD25 (BC96)	153Eu_CD25 (BC96)	173Yb_HLA-DR (LN3)
147Sm_CD11c (Bu15)	171Er_CD20 (2H7)	163Dy_CD56/NCAM (NCAM16.2)	154Sm_CD27 (O323)	154Sm_CD27 (O323)	155Gd_CD57 (HCD57)
148Nd_CD16 (3G8)	144Nd_CD19 (HIB19)	164Dy_TCRgd (B1)	160Gd_CD28 (CD28.2)	160Gd_CD28 (CD28.2)	175Lu_CD279/PD-1 (EH12.2H7)
149Sm_CD45RO (UCHL1)	148Nd_CD16 (3G8)	148Nd_CD16 (3G8)	173Yb_HLA-DR (LN3)	161Dy_CD38 (HB-7)	166Er_CD294 (BM16)
150Nd_CD45RA (HI100)	168Er_CD14 (63D3)	168Er_CD14 (63D3)	175Lu_CD279/PD-1 (EH12.2H7)	173Yb_HLA-DR (LN3)	151Eu_CD161 (HP-3G10)
151Eu_CD161 (HP-3G10)	173Yb_HLA-DR (LN3)	173Yb_HLA-DR (LN3)	155Gd_CD57 (HCD57)	175Lu_CD279/PD-1 (EH12.2H7)	154Sm_CD27 (O323)
152Sm_CD194/CCR4 (L291H4)	172Yb_CD66b (G10F5)	172Yb_CD66b (G10F5)	176Yb_CD127 (A019D5)	176Yb_CD127 (A019D5)	160Gd_CD28 (CD28.2)
153Eu_CD25 (BC96)	209Bi_CD11b/Mac-1 (ICRF44)	209Bi_CD11b/Mac-1 (ICRF44)	161Dy_CD38 (HB-7)	163Dy_CD56/NCAM (NCAM16.2)	169Er_CD33 (WM53)
154Sm_CD27 (O323)	147Sm_CD11c (Bu15)	147Sm_CD11c (Bu15)	151Eu_CD161 (HP-3G10)	148Nd_CD16 (3G8)	153Eu_CD25 (BC96)
155Gd_CD57 (HCD57)	166Er_CD294 (BM16)	151Eu_CD161 (HP-3G10)	142Nd_CD181/CXCR1 (8F1/CXCR1)	151Eu_CD161 (HP-3G10)	176Yb_CD127 (A019D5)
156Gd_CD183/CXCR3 (G025H7)	169Er_CD33 (WM53)	155Gd_CD57 (HCD57)	156Gd_CD183/CXCR3 (G025H7)	155Gd_CD57 (HCD57)	149Sm_CD45RO (UCHL1)
158Gd_CD185/CXCR5 (J252D4)	161Dy_CD38 (HB-7)	162Dy_CD80/B7.1 (2D10.4)	158Gd_CD185/CXCR5 (J252D4)	142Nd_CD181/CXCR1 (8F1/CXCR1)	150Nd_CD45RA (HI100)
159Tb_CD22 (HIB22)	143Nd_CD123 (6H6)	159Tb_CD22 (HIB22)	152Sm_CD194/CCR4 (L291H4)	156Gd_CD183/CXCR3 (G025H7)	142Nd_CD181/CXCR1 (8F1/CXCR1)
160Gd_CD28 (CD28.2)		154Sm_CD27 (O323)	141Pr_CD196/CCR6 (G034E3)	158Gd_CD185/CXCR5 (J252D4)	156Gd_CD183/CXCR3 (G025H7)
161Dy_CD38 (HB-7)		160Gd_CD28 (CD28.2)	167Er_CD197/CCR7 (G043H7)	152Sm_CD194/CCR4 (L291H4)	158Gd_CD185/CXCR5 (J252D4)
162Dy_CD80/B7.1 (2D10.4)		167Er_CD197/CCR7 (G043H7)		141Pr_CD196/CCR6 (G034E3)	152Sm_CD194/CCR4 (L291H4)
163Dy_CD56/NCAM (NCAM16.2)		149Sm_CD45RO (UCHL1)		167Er_CD197/CCR7 (G043H7)	141Pr_CD196/CCR6 (G034E3)
164Dy_TCRgd (B1)		150Nd_CD45RA (HI100)			167Er_CD197/CCR7 (G043H7)
165Ho_CD163 (GHI/61)		169Er_CD33 (WM53)			
166Er_CD294 (BM16)		161Dy_CD38 (HB-7)			
167Er_CD197/CCR7 (G043H7)		153Eu_CD25 (BC96)			
168Er_CD14 (63D3)		176Yb_CD127 (A019D5)			
169Er_CD33 (WM53)					
170Er_CD3 (UCHT1)					
171Er_CD20 (2H7)					
172Yb_CD66b (G10F5)					
173Yb_HLA-DR (LN3)					
174Yb_IgD (IA6-2)					
175Lu_CD279/PD-1 (EH12.2H7)					
176Yb_CD127 (A019D5)					
209Bi_CD11b/Mac-1 (ICRF44)					

Supplementary Table 4: Imaging Mass Cytometry (HYPERION) antibody information

Antibody	Clone	Metal	Reference	Dilution
CD14	5A3B11B5	141Pr	sc-58951	1/50
CD204	Polyclonal Goat IgG	142Nd	AF2708	1/200
DC LAMP	1010E1.01	144Nd	DDX0191P-100	1/150
TIGIT 1	E5Y1W	145Nd	17046SF	1/25
CD16	EPR16784	146Nd	3146020D	1/50
CD163	EDHu-1	147Sm	3147021D	1/200
Pan-Keratin	C11	148Nd	3148020D	1/200
Ki67	MKI67/2462	149Sm	ab237863	1/500
CD45	D9M8I	152Sm	3152018D	1/200
FoxP3	PCH101	155Gd	3155018D	1/100
CD4	EPR6855	156Gd	3156033D	1/200
DC-Sign	102E11.06	158Gd	DDX0202P	1/100
CD68	KP1	159Tb	3159035D	1/400
CD20	H1	161Dy	3161029D	1/400
CD8a	D8A8Y	162Dy	3162035D	1/300
CD138 (syndecan1)	SP152	163Dy	ab242394	1/200
MPO	EPR20257	164Dy	ab221847	1/1000
PD-1	D4W2J	165Ho	63815SF	1/100
CD56 (NCAM1)	NCAM1/1496	166Er	ab218925	1/50
GzB	EPR20129-217	167Er	3167021D	1/200
PD-L1	E1L3N	168Er	85164	1/100
Collagen Type I	Polyclonal	169Tm	3169023D	1/500
CD3	Polyclonal, C-Terminal	170Er	3170019D	1/200
CD206	685645	171Yb	MAB25341	1/100
Caspase-3 cleaved	5A1E	172Yb	3172027D	1/100
HLA DR	LN3	174Yb	3174023D	1/600
CD11c	ITGAX/1242	194Pt	ab212508	1/100
CD15	SP159	195Pt	ab240092	1/100
SMA	EPR5368	198Pt	ab220795	1/250

Supplementary Table 5: Clusters of innate and adaptive immune cells based on the expression of specific markers

Cell type	Subtypes	Phenotype
$CD4^{+}$T cells	Effector	$CD45RA^{+}$ $CCR7^{-}$
	EM Th1	$CD45RA^{-}$ $CCR7^{-}$ $CXCR3^{+}$ $CXCR5^{-}$ $CCR4^{-}$ $CCR6^{-}$
	EM Th17	$CD45RA^{-}$ $CCR7^{-}$ $CXCR3^{-}$ $CXCR5^{-}$ $CCR4^{+}$ $CCR6^{+}$
	CM Th1	$CD45RA^{-}$ $CCR7^{+}$ $CXCR3^{+}$ $CXCR5^{-}$ $CCR4^{-}$ $CCR6^{-}$
	CM Th1/Th17	$CD45RA^{-}$ $CCR7^{+}$ $CXCR3^{+}$ $CXCR5^{-}$ $CCR4^{+}$ $CCR6^{+}$
	CM Th17	$CD45RA^{-}$ $CCR7^{+}$ $CXCR3^{-}$ $CXCR5^{-}$ $CCR4^{+}$ $CCR6^{+}$
	CM Tfh	$CD45RA^{-}$ $CCR7^{+}$ $CXCR3^{-}$ $CXCR5^{+}$ $CCR4^{-}$ $CCR6^{-}$
	Treg	$CD25^{high}$ $CD127^{-}$
	Naive	$CD45RA^{+}$ $CD45RO^{-}$ $CCR7^{+}$
	Naive-CM/effector	$CD45RA^{+}$ $CD45RO^{+}$ $CCR7^{+}$
$CD8^{+}$T cells	Effector	$CD45RA^{+}$ $CCR7^{-}$
	EM	$CD45RA^{-}$ $CCR7^{-}$
	CM	$CD45RA^{-}$ $CCR7^{+}$
	Naive	$CD45RA^{+}$ $CCR7^{+}$
	Naive-CM/effector	$CD45RA^{+}$ $CD45RO^{+}$ $CCR7^{+}$
	NKT	$CD56^{+}$ $CD161^{-}$
	NKT/MAIT	CD56dim $CD161^{+}$ $CD57^{-}$
	MAIT	$CD56^{-}$ $CD161^{+}$ $CD57^{-}$
NK cells	$NKCD56^{-}$ $HLA\text{-}DR^{+}$	$CD8^{low}$ $CD56^{-}$ $CD16^{+}$ $HLA\text{-}DR^{+}$ $CD57^{-}$
	$NKCD56^{dim}$	$CD56^{dim}$ $CD16^{+}$
	$NKCD56^{bright}$	$CD56^{bright}$ $CD16^{low}$ $CD57^{-}$
$CD4^{-}CD8^{-}$T cells	γδ T cells	$CD4^{-}$ $CD8^{-}$ $TCR\gamma\delta^{+}$
Monocytes	Classical monocytes	$CD4^{+}$ $CD14^{high}$ $CD16^{low}$ $HLA\text{-}DR^{low}$
	Intermediate monocytes	$CD4^{+}$ $CD14^{dim}$ $CD16^{dim}$ $HLA\text{-}DR^{dim}$
	Non-classical monocytes	$CD4^{+}$ $CD14^{low}$ $CD16^{high}HLA\text{-}DR^{high}$
Other cell types	B cells	$CD19^{+}$ $CD20^{+}$
	Dendritic cells	$CD14^{-}$ $CD4^{+}$ $HLA\text{-}DR^{+}$ $CD11c^{+}$ $CD33^{+}$
	Neutrophils	$CD14^{-}$ $CD16^{+}$ $HLA\text{-}DR^{-}$ $CD66b^{+}$ $CD11b^{+}$ $CD11c^{+}$
	Eosinophils	$CD14^{-}$ $HLA\text{-}DR^{+}$ $CD294^{dim}$ $CD123^{high}$
	Basophils	$CD14^{-}$ $HLA\text{-}DR^{-}$ $CD294^{high}$ $CD123^{dim}$

Supplementary Table 6: Ingenuity pathway analysis identifies major upstream regulators of eicosanoids. Regulators are classified in descending B-H (Benjamini-Hochberg) corrected p-value

Regulator	Molecule type	B-H corrected p-value	Target molecules
Zymosan	Chemical – endogenous non-mammalian	2.87E-10	15-HETE, 5-HETE, Dinoprost, LTB4, PGD2, PGE2, TXB2
TNF	Cytokine	0.00000114	5-HETE, Dinoprost, LTB4, PGD2, PGE2, TXB2
Arachidonic acid	Chemical – endogenous mammalian	0.00000182	15-HETE, Dinoprost, LTB4, PGE2, TXB2
IL4	Cytokine	0.0000335	15-HETE, Dinoprost, LTB4, PGD2, PGE2, TXB2
CXCL1	Cytokine	0.0000363	LTB4, PGD2, PGE2, TXB2
CSF2	Cytokine	0.000115	LTB4, PGE2
IL1B	Cytokine	0.000199	5-HETE, LTB4, PGE2
EDN1	Cytokine	0.0003	Dinoprost, PGD2, PGE2, TXB2
CD40LG	Cytokine	0.000391	Dinoprost, PGD2, PGE2
IL18	Cytokine	0.000932	Dinoprost, PGE2
IL1RN	Cytokine	0.0012	LTB4, PGE2
CXCL8	Cytokine	0.00223	PGE2, TXB2
IL2	Cytokine	0.0025	LTB4, PGE2
C5	Cytokine	0.00337	PGE2, TXB2
IL10	Cytokine	0.0038	LTB4, PGE2
WNT1	Cytokine	0.00529	PGE2, TXB2
PF4	Cytokine	0.00795	PGE2
IL9	Cytokine	0.00795	TXB2
IL27	Cytokine	0.00795	PGE2
CXCL3	Cytokine	0.0126	PGE2
CCL3L3	Cytokine	0.0157	LTB4
IL15	Cytokine	0.0157	LTB4
IL5	Cytokine	0.0192	PGE2
OSM	Cytokine	0.0192	PGD2
TNFSF10	Cytokine	0.0228	PGE2
CNTF	Cytokine	0.0261	PGE2
CCL2	Cytokine	0.0261	PGE2
MIF	Cytokine	0.0261	LTB4
IL3	Cytokine	0.0314	PGE2
CSF3	Cytokine	0.0345	PGD2
IL1A	Cytokine	0.0407	PGE2

Supplementary Table 7: Antibodies used in flow cytometer, functional studies, and SCENITH experiments

Antibody	Clone	Source	Cat# Identifier
CD3-BUV805	UCTH1	BD Bioscience	612895
CD4-APCH7	RPA-T4	BD Bioscience	560158
CD8-BV421	RPA-T8	BD Bioscience	562428
IFNγ-BV650	B27	BD Bioscience	557643
TNFα-PeCy7	MP6-XT22	BD Bioscience	563943
PD1-FITC	EH12-1	BD Bioscience	564494
LAG3-PE	T47-530	BD Bioscience	565616
CD56-APC	REA196	Miltenyi Biotec	130-113-310
CD107-PeCy7	REA792	Miltenyi Biotec	130-111-622
NKG2D-APCVio770	REA797	Miltenyi Biotec	130-129-851
NKG2A-FITC	REA110	Miltenyi Biotec	130-113-565
NKG2C- PEVio615	REA205	Miltenyi Biotec	130-123-047
KIR2D-VioGreen	REA1042	Miltenyi Biotec	130-117-630
CD8-BV605	RPA-T8	Biolegend	301040
CD4-BUV496	SK3	BD Bioscience	612936
CD3-APC-Vio770	REA613	Miltenyi Biotec	130-113-136
CD56-BV650	NCAM16.2	BD Bioscience	564057
CD16-BUV395	3G8	BD Bioscience	563785
CD14-BV570	MSE2	Biolegend	301832
