# Supplementary material for: Systems immunology integrates the complex endotypes of recessive dystrophic epidermolysis bullosa
Source: Nat Commun. 2025 Jan 14;16:664. doi: 10.1038/s41467-025-55934-7 (PMC11733305; doi:10.1038/s41467-025-55934-7)
Supplement: Supplementary file 2 — Reporting Summary [file 41467_2025_55934_MOESM2_ESM.pdf]

nature portfolio

Corresponding author(s): Reem Al-Daccak

Last updated by author(s): 30/09/24

Reporting Summary

Nature Portfolio wishes to improve the reproducibility of the work that we publish. This form provides structure for consistency and transparency in reporting. For further information on Nature Portfolio policies, see our Editorial Policies and the Editorial Policy Checklist.

Statistics

For all statistical analyses, confirm that the following items are present in the figure legend, table legend, main text, or Methods section.

n/a	Confirmed	
☐	☒	The exact sample size (*n*) for each experimental group/condition, given as a discrete number and unit of measurement
☐	☒	A statement on whether measurements were taken from distinct samples or whether the same sample was measured repeatedly
☐	☒	The statistical test(s) used AND whether they are one- or two-sided<br>*Only common tests should be described solely by name; describe more complex techniques in the Methods section.*
☐	☒	A description of all covariates tested
☒	☐	A description of any assumptions or corrections, such as tests of normality and adjustment for multiple comparisons
☐	☒	A full description of the statistical parameters including central tendency (e.g. means) or other basic estimates (e.g. regression coefficient) AND variation (e.g. standard deviation) or associated estimates of uncertainty (e.g. confidence intervals)
☐	☒	For null hypothesis testing, the test statistic (e.g. *F*, *t*, *r*) with confidence intervals, effect sizes, degrees of freedom and *P* value noted<br>*Give P values as exact values whenever suitable.*
☒	☐	For Bayesian analysis, information on the choice of priors and Markov chain Monte Carlo settings
☒	☐	For hierarchical and complex designs, identification of the appropriate level for tests and full reporting of outcomes
☒	☐	Estimates of effect sizes (e.g. Cohen's *d*, Pearson's *r*), indicating how they were calculated

Our web collection on statistics for biologists contains articles on many of the points above.

Software and code

Policy information about availability of computer code

Data collection	OMIQ (Dotmatics)
Data analysis	Mass 19 Hunter quantitative, R-Studio, Ingenuity Pathway analysis (Qiagen), GraphPad (Prism9.5)

For manuscripts utilizing custom algorithms or software that are central to the research but not yet described in published literature, software must be made available to editors and reviewers. We strongly encourage code deposition in a community repository (e.g. GitHub). See the Nature Portfolio guidelines for submitting code & software for further information.

Data

Policy information about availability of data

All manuscripts must include a data availability statement. This statement should provide the following information, where applicable:

- Accession codes, unique identifiers, or web links for publicly available datasets
- A description of any restrictions on data availability
- For clinical datasets or third party data, please ensure that the statement adheres to our policy

All data supporting the findings of this study are available within the paper and its Supplementary Information.

Research involving human participants, their data, or biological material

Policy information about studies with human participants or human data. See also policy information about sex, gender (identity/presentation), and sexual orientation and race, ethnicity and racism.

Reporting on sex and gender	Blood samples from a small clinical-biological cohort of adult patients : 8 females, 4 males. Skin biopsies from 2 patients and 2 healthy individuals who underwent breast reduction plastic surgery. Sex is considered in the study design.
Reporting on race, ethnicity, or other socially relevant groupings	N/A
Population characteristics	Patients suffering from Recessive Dystrophic Epidermolysis Bullosa
Recruitment	Patients are followed and managed at the Dermatology Department – French national reference center for rare diseases of the skin and mucous membranes of genetic origin (MAGEC). Samples were taken outside of any clinical opportunistic infection or active cancer.
Ethics oversight	Study approuved by the National committee “Comité consultatif pour la protection des personnes dans les recherches biomédicales” AP-HP, Saint-Louis Hospital, Paris, France. REC-DTYP-0438

Note that full information on the approval of the study protocol must also be provided in the manuscript.

Field-specific reporting

Please select the one below that is the best fit for your research. If you are not sure, read the appropriate sections before making your selection.

☒ Life sciences ☐ Behavioural & social sciences ☐ Ecological, evolutionary & environmental sciences

For a reference copy of the document with all sections, see nature.com/documents/nr-reporting-summary-flat.pdf

Life sciences study design

All studies must disclose on these points even when the disclosure is negative.

Sample size	12 patients and 9 Healthy donors
Data exclusions	N/A
Replication	At least 3 replicates for each data set
Randomization	N/A
Blinding	N/A

Reporting for specific materials, systems and methods

We require information from authors about some types of materials, experimental systems and methods used in many studies. Here, indicate whether each material, system or method listed is relevant to your study. If you are not sure if a list item applies to your research, read the appropriate section before selecting a response.

Materials & experimental systems

n/a	Involved in the study	
☐	☒	Antibodies
☐	☒	Eukaryotic cell lines
☒	☐	Palaeontology and archaeology
☒	☐	Animals and other organisms
☐	☒	Clinical data
☐	☒	Dual use research of concern
☒	☐	Plants

Methods

n/a	Involved in the study	
☒	☐	ChIP-seq
☐	☒	Flow cytometry
☒	☐	MRI-based neuroimaging

Antibodies

Antibodies used	All antibodies are listed in Supplementary Information under Table 3, 4 and 7

Validation	The specificity and concentration for all used antibodies were validated prior experiments.

Eukaryotic cell lines

Policy information about cell lines and Sex and Gender in Research

Cell line source(s)	Human Lymphoblast cell line (K562)
Authentication	ATCC: CCL-243
Mycoplasma contamination	Cell line were tested mycoplasma free on regular basis.
Commonly misidentified lines (See ICLAC register)	N/A

Clinical data

Policy information about clinical studies

All manuscripts should comply with the ICMJEguidelines for publication of clinical research and a completedCONSORT checklist must be included with all submissions.

Clinical trial registration	N/A
Study protocol	*Note where the full trial protocol can be accessed OR if not available, explain why.*
Data collection	*Describe the settings and locales of data collection, noting the time periods of recruitment and data collection.*
Outcomes	*Describe how you pre-defined primary and secondary outcome measures and how you assessed these measures.*

Plants

Seed stocks	N/A
Novel plant genotypes	N/A
Authentication	N/A

Flow Cytometry

Plots

Confirm that:

- [x] The axis labels state the marker and fluorochrome used (e.g. CD4-FITC).
- [x] The axis scales are clearly visible. Include numbers along axes only for bottom left plot of group (a 'group' is an analysis of identical markers).
- [x] All plots are contour plots with outliers or pseudocolor plots.
- [x] A numerical value for number of cells or percentage (with statistics) is provided.

Methodology

Sample preparation	Fresh whole blood samples were collected in sodium heparinized tubes and immune cell subsets were determined by flow-cytometry. Skin biopsies were collected, fixed in formalin, and embedded in paraffin, then section were subjected to imaging mass cytometry.
Instrument	CyTOF XT Mass Cytometer, Fortessa, Canto II, Symphony A5, Hyperion Imaging System™
Software	OMIQ, FlowJo software (10.8.1), YOUPI software, MCD™ Viewer 1.0
Cell population abundance	The abundance of all cell populations is described in figure legends.

Gating strategy	Gating strategy is detailed in Supplementary Fig. 1a and Fig. 8.

☒ Tick this box to confirm that a figure exemplifying the gating strategy is provided in the Supplementary Information.
